# Variation in surgical demand and time to hip fracture repair: a Canadian database study

**DOI:** 10.1186/s12913-020-05791-5

**Published:** 2020-10-10

**Authors:** Katie J. Sheehan, Boris Sobolev, Pierre Guy, Jason D. Kim, Lisa Kuramoto, Lauren Beaupre, Adrian R. Levy, Suzanne N. Morin, Jason M. Sutherland, Edward J. Harvey, for the Canadian Collaborative Study on Hip Fractures, Lauren Beaupre, Eric Bohm, Michael Dunbar, Donald Griesdale, Pierre Guy, Edward Harvey, Erik Hellsten, Susan Jaglal, Hans Kreder, Lisa Kuramoto, Adrian Levy, Suzanne N. Morin, Katie J. Sheehan, Boris Sobolev, Jason M. Sutherland, James Waddell

**Affiliations:** 1 Department of Population Health Sciences, School of Population Health and Environmental Sciences, Faculty of Life Science and Medicine, King’s College London, London, UK; 2 School of Population and Public Health, University of British Columbia, Vancouver, British Columbia Canada; 3 Centre for Hip Health and Mobility, University of British Columbia, Vancouver, British Columbia Canada; 4 Centre for Clinical Epidemiology & Evaluation, Vancouver Coastal Health Research Institute, University of British Columbia, Vancouver, British Columbia Canada; 5 Department of Physical Therapy and Division of Orthopaedic Surgery, University of Alberta, Edmonton, Alberta Canada; 6 Department of Community Health and Epidemiology, Dalhousie University, Halifax, Nova Scotia Canada; 7 Department of Medicine, McGill University, Montreal, Quebec Canada; 8 Centre for Health Services and Policy Research, School of Population and Public Health, University of British Columbi, Vancouver, British Columbia Canada; 9 Division of Orthopaedic Surgery, McGill University, Montreal, Canada

**Keywords:** Hip fracture, Time to surgery, Surgical delay, Demand, Variation

## Abstract

**Background:**

Competing demands for operative resources may affect time to hip fracture surgery. We sought to determine the time to hip fracture surgery by variation in demand in Canadian hospitals.

**Methods:**

We obtained discharge abstracts of 151,952 patients aged 65 years or older who underwent surgery for a hip fracture between January, 2004 and December, 2012 in nine Canadian provinces. We compared median time to surgery (in days) when demand could be met within a two-day benchmark and when demand required more days, i.e. clearance time, to provide surgery, overall and stratified by presence of medical reasons for delay.

**Results:**

For persons admitted when demand corresponded to a 2-day clearance time, 68% of patients underwent surgery within the 2-day benchmark. When demand corresponded to a clearance time of one week, 51% of patients underwent surgery within 2 days. Compared to demand that could be served within the two-day benchmark, adjusted median time to surgery was 5.1% (95% confidence interval [CI] 4.1–6.1), 12.2% (95% CI 10.3–14.2), and 22.0% (95% CI 17.7–26.2) longer, when demand required 4, 6, and 7 or more days to clear the backlog, respectively. After adjustment, delays in median time to surgery were similar for those with and without medical reasons for delay.

**Conclusion:**

Increases in demand for operative resources were associated with dose-response increases in the time needed for half of hip fracture patients to undergo surgery. Such delays may be mitigated through better anticipation of day-to-day supply and demand and increased response capability.

## Background

Outcomes of hip fractures are poor, with up to 10% of patients dying in hospital and 30% within one year [1, 2]. Poor outcomes have been attributed to patient characteristics and their injury, and less frequently, to their care [3]. Most patients undergo surgery to treat their hip fracture [4]. Access to care can be characterized by the timely delivery of appropriate care to achieve the best health outcomes [5]. Indeed, expeditious surgical repair is shown to reduce the risk of major perioperative complications, 6% vs 8%, [6] and inhospital death, 5% vs 7% [7].

However, the timely use of hip fracture surgery continues to be suboptimal in several countries [8]. For example, almost one-third of patients with no medical reasons for delay wait longer than the two-day benchmark established by Canada’s federal, provincial, and territorial governments [7]. At the hospital level, time to surgery is governed by unpredictable variation in demand for hip repair, the number of patients already awaiting surgery, the time needed to provide care, [9] as well as competition for the same operative resources with other non-scheduled patients [10]. Medically unwarranted delays ensue when competing demands mismatch available capacity [11].

Currently, there is no empirical evidence detailing the extent to which variation in demand contributes to surgical delays after a hip fracture. This knowledge is important when informing optimum capacity for treating this vulnerable patient population within the recommended benchmark. Therefore, we sought to compare the time to surgery after hip fracture, between those admitted when demand can be met within two days (benchmark demand), 4 days (medium demand), 6 days (high demand), and 7 or more days (excessive demand), overall and by the presence of a medical reason for delay.

## Methods

### Design, setting, and population

We obtained population-based discharge abstracts of 154,389 patients 65 years or older surgically-treated for non-pathological first hip fracture between January 1, 2004 and December 31, 2012 in all Canadian hospitals, except those in Quebec, from the Canadian Institute for Health Information (CIHI) [12]. Multiple abstracts with the same patient identifier and contiguous dates of discharge and admission were combined into one care episode using the CIHI hospital transfers rules [12]. We excluded 2437 patients surgically-treated in a hospital with an annual volume of 24 hip fracture surgeries or less (this included all surgeries performed in the three northern Territories), [13] leaving 151,952 patients for analysis. The University of British Columbia Behavioural Research Ethics Board approved this study (H11–02611).

### Outcome

The primary outcome was time to surgery determined by the dates of admission and surgery. Hospital stay was measured as the number of inpatient days. The censoring interval for time to surgery was [N - 1, N], where N is the inpatient day on which surgery was performed. We right censored observations at seven days considering delays beyond this time to be due to medical instability rather than resource demand [14]. We took the natural logarithm of the interval bounds to fit a log-normal model, a standard approach.

### Study variable

We classified demand according to the time it would take all concurrently hospitalized patients with a hip fracture to undergo surgery if there were no new arrivals (clearance time): within 2 days (benchmark demand), 4 days (medium demand), 6 days (high demand), and 7 or more days (excessive demand) (Table 1) [15]. For each patient, we estimated the clearance time by dividing the number of preoperative patients with a hip fracture present in hospital on the day of admission by the maximum weekly service rate at the same hospital in the corresponding fiscal quarter. The maximum rate estimates the largest number of surgeries a hospital can deliver during a quarter, thereby allowing to estimate the hospital’s capacity to manage the existing demand. We expressed the clearance time in days by dividing the maximum weekly rate by seven. The maximum rate was quarter- and hospital- specific to account for variation in the allocation of resources to hip fracture care over time.

**Table 1:** Key concepts for the study variable

Concept	Definition
Clearance time	The expected length of time within which all patients hospitalized for hip fracture could undergo surgery when hospital operates at a maximum weekly service rate if there were no new arrivals.
Maximum weekly service rate	The maximum number of patients undergoing hip fracture surgery in a single week during a fiscal quarter for a given hospital.
Benchmark demand	All hospitalized patients could undergo surgery within two days.
Medium demand	All hospitalized patients could undergo surgery within 4 days.
High demand	All hospitalized patients could undergo surgery within 6 days.
Excessive demand	All hospitalized patients require 7 or more days to undergo surgery.

### Subgroups

We defined subgroups by the presence/absence of a medical reason for delay. Medical reasons for delay were identified as at least one preoperative specialist-care-unit (SCU) admission (e.g. intensive care unit, coronary care unit) or, at least one of the National Institute for Health and Care Excellence guideline-124 (NICE-124) conditions that may delay surgery if not treated promptly [16]. These conditions include anaemia, anticoagulation, volume depletion, electrolyte imbalance, uncontrolled diabetes, uncontrolled heart failure, acute cardiac arrhythmia or ischemia, acute chest infection or exacerbation of a chronic chest condition. We previously demonstrated 6.7% of patients with a hip fracture present with at least one of these conditions [17].

### Statistical analysis

We wrote a statistical analysis plan before undertaking analyses (Supplementary File 1). We report patient, injury, and care characteristics by demand status as frequencies and percentages. We used parametric interval regression to estimate the median time to surgery in relation to level of demand: medium, high, excessive, as compared to the two-day benchmark [18]. We treated time to surgery as right-censored observations if surgery was performed after 7 inpatient days, and as interval censored observations if performed on any other inpatient day. We modeled the natural logarithm of the outcome and included random intercepts for hospital and random coefficients for demand. The shape of the empirical distribution function of surgery by time from admission corresponded to a lognormal distribution. Standard errors of the regression coefficients were estimated by a clustered sandwich estimator.

We report the percentage change in the median time to surgery between patients admitted when demand was greater than the 2-day benchmark (within 4 days, 6 days, 7 or more days) and patients admitted when demand was within the 2-day benchmark, adjusting for age, sex, prefracture health status, [19, 20] admission timing, admission status (urgent/emergency, otherwise), preoperative transfer, preoperative procedures, medical reason for delay, hospital type, fracture type (transcervical, intertrochanteric/subtrochanteric), type of surgery (fixation, arthroplasty), treatment era (2004–2006, 2007–2009, 2010–2012), and province. In addition, we estimated the cumulative probabilities of surgery within certain times since admission when demand was greater than the 2-day benchmark and when demand was within the 2-day benchmark using a non-parametric method for interval-censored data. Stata 15 was used for analyses [21].

## Results

### Patients characteristics

Almost half of patients were 85 years of age or older (45.7%), admitted from home without comorbidity (42.5%), admitted with a transcervical hip fracture (52.1%), underwent fixation (59.8%), and were treated in Ontario (48.5%)(Table 2). On average, hospitals treated 190 (interquartile interval: 131–237) hip fractures each year. Overall, 61,090 patients (40.2%) were admitted when demand was within the 2-day benchmark, 71,183 (46.9%) within 4 days, 17,192 (11.3%) within 6 days, and 2487 (1.6%) 7 days or more. Most patients presented without a recorded medical reason for delay (93.3%).

**Table 2:** Characteristics of 151,952 patients who underwent surgery for first hip fracture in Canada, 2004–2012, overall and by demand

			Demand, by clearance time^a^; no. (%) of patients							
	All patients(*N* = 151,952)		Benchmark demand(*N* = 61,090)		Medium demand(*N* = 71,183)		High demand(*N* = 17,192)		Excessive demand (*N* = 2487)	
	n	(%)	n	(%)	n	(%)	n	(%)	n	(%)
Age, years										
65–74	22,859	(15.0)	9039	(14.8)	10,755	(15.1)	2646	(15.4)	419	(16.8)
75–84	59,688	(39.3)	23,899	(39.1)	27,970	(39.3)	6829	(39.7)	990	(39.8)
85–94	61,415	(40.4)	24,866	(40.7)	28,739	(40.4)	6850	(39.8)	960	(38.6)
≥ 95	7990	(5.3)	3286	(5.4)	3719	(5.2)	867	(5.0)	118	(4.7)
Sex^b^										
Women	111,560	(73.4)	44,737	(73.2)	52,463	(73.7)	12,579	(73.2)	1781	(71.6)
Men	40,375	(26.6)	16,345	(26.8)	18,712	(26.3)	4612	(26.8)	706	(28.4)
Prefracture health: from^c^										
Home without comorbidity	64,594	(42.5)	25,789	(42.2)	30,234	(42.5)	7486	(43.5)	1085	(43.6)
Home with comorbidity or home care	27,467	(18.1)	10,895	(17.8)	12,870	(18.1)	3213	(18.7)	489	(19.7)
Facility	31,644	(20.8)	12,864	(21.1)	14,725	(20.7)	3522	(20.5)	533	(21.4)
Elsewhere	28,247	(18.6)	11,542	(18.9)	13,354	(18.8)	2971	(17.3)	380	(15.3)
Timing of admission										
Weekday 12 am to 3:59 pm	51,797	(34.1)	20,809	(34.1)	24,161	(33.9)	5991	(34.8)	836	(33.6)
Weekday 4 pm to 11:59 pm	57,894	(38.1)	23,274	(38.1)	27,293	(38.3)	6410	(37.3)	917	(36.9)
Weekend	42,261	(27.8)	17,007	(27.8)	19,729	(27.7)	4791	(27.9)	734	(29.5)
Admission status										
Urgent/Emergency	149,212	(98.2)	59,994	(98.2)	69,901	(98.2)	16,874	(98.2)	2443	(98.2)
Otherwise	2740	(1.8)	1096	(1.8)	1282	(1.8)	318	(1.8)	44	(1.8)
Preoperative transfer history										
No	138,836	(91.4)	55,714	(91.2)	64,983	(91.3)	15,839	(92.1)	2300	(92.5)
Yes	13,116	(8.6)	5376	(8.8)	6200	(8.7)	1353	(7.9)	187	(7.5)
Preoperative procedures										
No	135,077	(88.9)	54,447	(89.1)	63,318	(89.0)	15,166	(88.2)	2146	(86.3)
Yes	16,875	(11.1)	6643	(10.9)	7865	(11.0)	2026	(11.8)	341	(13.7)
Medical reason for delay^d^										
No	141,808	(93.3)	57,136	(93.5)	66,376	(93.2)	15,986	(93.0)	2310	(92.9)
Yes	10,144	(6.7)	3954	(6.5)	4807	(6.8)	1206	(7.0)	177	(7.1)
Hospital type at surgery^e^										
Teaching	59,281	(39.0)	23,574	(38.6)	29,105	(40.9)	6031	(35.1)	571	(23.0)
Community-Large	69,597	(45.8)	29,132	(47.7)	31,378	(44.1)	8179	(47.6)	908	(36.5)
Community-Medium, Small	21,508	(14.2)	7713	(12.6)	10,057	(14.1)	2779	(16.2)	959	(38.6)
Fracture type										
Transcervical	79,127	(52.1)	31,897	(52.2)	37,126	(52.2)	8760	(51.0)	1344	(54.0)
Intertrochanteric or subtrochanteric	72,825	(47.9)	29,193	(47.8)	34,057	(47.8)	8432	(49.0)	1143	(46.0)
Procedure type										
Fixation	90,852	(59.8)	36,515	(59.8)	42,464	(59.7)	10,420	(60.6)	1453	(58.4)
Arthroplasty	61,100	(40.2)	24,575	(40.2)	28,719	(40.3)	6772	(39.4)	1034	(41.6)
Treatment era										
2004–2006	50,627	(33.3)	20,148	(33.0)	23,670	(33.3)	5962	(34.7)	847	(34.1)
2007–2009	50,157	(33.0)	19,550	(32.0)	23,921	(33.6)	5760	(33.5)	926	(37.2)
2010–2012	51,168	(33.7)	21,392	(35.0)	23,592	(33.1)	5470	(31.8)	714	(28.7)
Province of surgery										
Alberta	16,644	(11.0)	7440	(12.2)	7726	(10.9)	1363	(7.9)	115	(4.6)
British Columbia	28,922	(19.0)	11,762	(19.3)	13,731	(19.3)	3086	(18.0)	343	(13.8)
Manitoba	8439	(5.6)	2880	(4.7)	3996	(5.6)	1364	(7.9)	199	(8.0)
New Brunswick	5285	(3.5)	1834	(3.0)	2454	(3.4)	789	(4.6)	208	(8.4)
Newfoundland and Labrador	3435	(2.3)	1110	(1.8)	1746	(2.5)	479	(2.8)	100	(4.0)
Nova Scotia	6556	(4.3)	2157	(3.5)	3325	(4.7)	899	(5.2)	175	(7.0)
Ontario	73,629	(48.5)	30,310	(49.6)	33,742	(47.4)	8369	(48.7)	1208	(48.6)
Prince Edward Island	1100	(0.7)	500	(0.8)	456	(0.6)	136	(0.8)	8	(0.3)
Saskatchewan	7942	(5.2)	3097	(5.1)	4007	(5.6)	707	(4.1)	131	(5.3)

^a^The expected length of time for all hospitalized patients with hip fracture present on day of index patient admission to undergo surgery when hospital operates at maximum weekly service rate for the corresponding fiscal quarter. Benchmark demand (within 2-days), medium demand (within 4 days), high demand (within 6 days), and excessive demand (7 or more days)

^b^For 17 patients, sex was unknown

^c^Comorbidities included heart failure, chronic obstructive pulmonary disorder, ischemic heart disease (acute and chronic), dysrhythmias, hypertension, diabetes, and cancer (breast–female, prostate, renal, lung, multiple myeloma, and metastatic cancer) identified by diagnostic codes from all hospitalizations in 1 year prior to index admission, and cancer and Paget’s disease, identified by diagnostic codes from all hospitalizations during the hip fracture care episode

^d^At least one of the following NICE-124 conditions: anaemia, anticoagulation reversal, volume depletion, electrolyte imbalance, uncontrolled diabetes, uncontrolled heart failure, correctable cardiac arrhythmia, correctable cardiac ischaemia, acute chest infection, and exacerbation of chronic chest condition; or preoperative admission to specialist care unit

^e^For 1566 patients, hospital type was unavailable

### Median time to surgery by demand

**Fig. 1:**
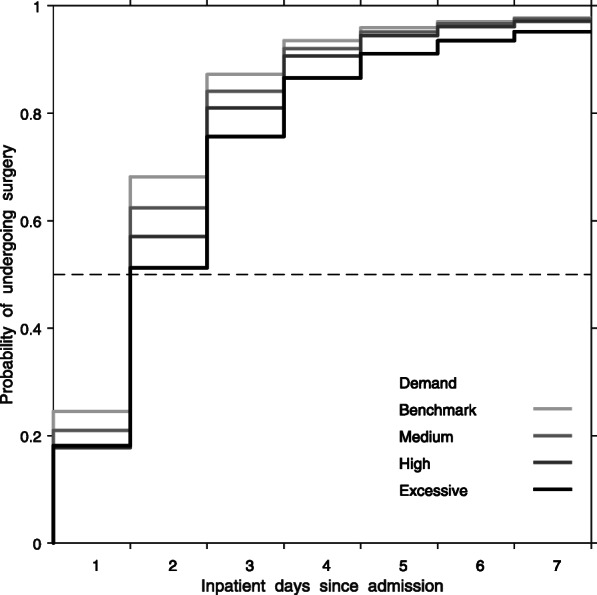
Unadjusted probability of undergoing surgery within a certain time, by demand. Demand is measured by clearance time, the expected length of time for all patients hospitalized with hip fracture present on day of admission to undergo surgery when hospital operates at maximum weekly service rate for the corresponding fiscal quarter. Benchmark demand (within 2-days), medium demand (within 4 days), high demand (within 6 days), and excessive demand (7 or more days)

For patients admitted when demand was within the 2-day benchmark, 68, 94, and 98% underwent surgery within 2, 4, and 7 days. For 71,183 patients admitted during medium demand, 62, 92, and 98% underwent surgery within 2, 4, and 7 days. For 17,192 patients admitted during high demand, 57, 91, and 97% underwent surgery within 2, 4, and 7 days. For 2487 patients admitted during excessive demand, 51, 87, and 95% underwent surgery within 2, 4, and 7 days (Fig. 1).

The median time to surgery when demand was within the 2-day benchmark was 1.5 days (95% confidence interval [CI] 1.5–1.6). The median time to surgery when demand required 4, 6, and 7 or more days to provide surgery was 1.7 days (95% CI 1.6–1.7), 1.8 days (95% CI 1.7–1.9), and 1.9 days (95% CI 1.8–2.1) (Table 3). After adjustment, median time to surgery was 5.1% (95% CI 4.1–6.1), 12.2% (95% CI 10.3–14.2), and 22.0% (95% CI 17.7–26.2) longer when demand required 4-, 6-, or 7 or more- days to provide surgery, compared to the 2-day benchmark.

**Table 3:** Time to surgery for first hip fracture by demand, overall and by the presence/absence of medical reasons for surgical delay

**Overall**				
Demand, by clearance time^a^	No. of Surgeries	Median Time to Surgery, Days (95% CI)	Unadjusted Percentage Change, % (95% CI)^b^	Adjusted Percentage Change, % (95% CI)^bcd^
Benchmark demand	61,090	1.5 (1.5 to 1.6)	Reference	Reference
Medium demand	71,183	1.7 (1.6 to 1.7)	8.6 (7.8 to 9.4)	5.1 (4.1 to 6.1)
High demand	17,192	1.8 (1.7 to 1.9)	17.2 (15.9 to 18.6)	12.2 (10.3 to 14.2)
Excessive demand	2487	1.9 (1.8 to 2.1)	27.0 (23.6 to 30.5)	22.0 (17.7 to 26.2)
**Without a medical reason for surgical delay**				
Demand, by clearance time^a^	No. of Surgeries	Median Time to Surgery, Days (95% CI)	Unadjusted Percentage Change, % (95% CI)^b^	Adjusted Percentage Change, % (95% CI)^b e d^
Benchmark demand	57,136	1.5 (1.4 to 1.5)	Reference	Reference
Medium demand	66,376	1.6 (1.5 to 1.7)	8.5 (7.7 to 9.3)	5.1 (4.1 to 6.1)
High demand	15,986	1.7 (1.7 to 1.8)	17.3 (15.9 to 18.6)	12.6 (10.6 to 14.5)
Excessive demand	2310	1.9 (1.8 to 2.0)	27.3 (23.8 to 30.8)	22.2 (17.8 to 26.6)
**With a medical reason for surgical delay**				
Demand, by clearance time^a^	No. of Surgeries	Median Time to Surgery, Days (95% CI)	Unadjusted Percentage Change, % (95% CI)^b^	Adjusted Percentage Change, % (95% CI)^b e d^
Benchmark demand	3954	2.2 (2.1 to 2.4)	Reference	Reference
Medium demand	4807	2.4 (2.3 to 2.5)	8.3 (4.6 to 12.0)	5.6 (2.0 to 9.3)
High demand	1206	2.5 (2.4 to 2.7)	12.4 (6.5 to 18.4)	9.1 (3.0 to 15.2)
Excessive demand	177	2.7 (2.2 to 3.1)	19.0 (4.2 to 33.7)	20.0 (1.8 to 38.1)

^a^The expected length of time for all hospitalized patients with hip fracture present on day of index patient admission to undergo surgery when hospital operates at maximum weekly service rate for the corresponding fiscal quarter. Benchmark demand (within 2-days), medium demand (within 4 days), high demand (within 6 days), and excessive demand (7 or more days)

^b^Percentage change is the median time to surgery in one demand group minus the median time to surgery in the reference group, divided by the median time in the reference group, and multiplied by 100%. It is estimated by subtracting one from the exponential of the regression coefficient and multiplying the result by 100%

^c^Adjusted for age, sex, prefracture health status, timing of admission, admission status, preoperative transfer history, preoperative procedures, medical reason for delay, hospital type at surgery, fracture type, procedure type, treatment era, and province

^d^Random intercepts by hospital ID and random coefficients for demand; random effects by grouping patients according to hospital ID and allowing the effect of demand to vary by the grouping structure

^e^ Adjusted for age, sex, prefracture health status, timing of admission, admission status, preoperative transfer history, preoperative procedures, hospital type at surgery, fracture type, procedure type, treatment era, and province

### Patients without a medical reason for delay

**Fig. 2:**
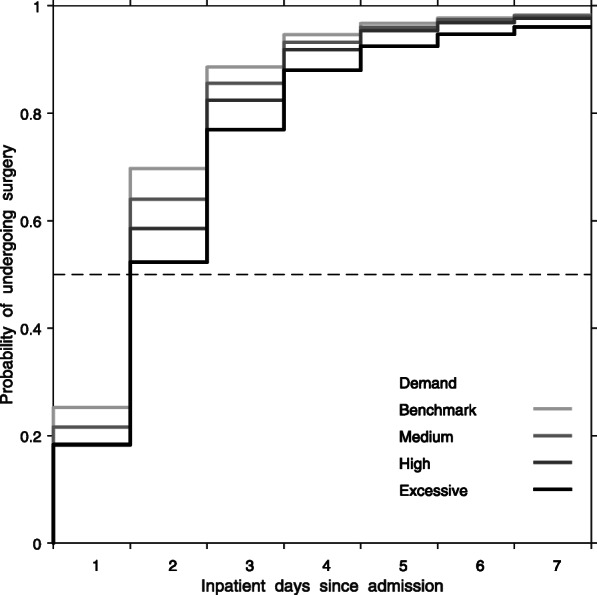
Unadjusted probability of undergoing surgery within a certain time, by demand, among those without a medical reason for delay. Benchmark demand (within 2-days), medium demand (within 4 days), high demand (within 6 days), and excessive demand (7 or more days)

Overall, 84,672 of 141,808 patients (59.7%) without a medical reason for delay were admitted when demand was greater than the 2-day benchmark. For 57,136 patients admitted when demand was within the 2-day benchmark, 70, 95, and 98% underwent surgery within 2, 4, and 7 days. For 66,376 patients admitted during medium demand, 64, 93, and 98% underwent surgery within 2, 4, and 7 days. For 15,986 patients admitted during high demand, 59, 92, and 98% underwent surgery within 2, 4, and 7 days. For 2310 patients admitted during excessive demand, 52, 88, and 96% underwent surgery within 2, 4, and 7 days (Fig. 2). The percentage change between excessive and benchmark demand reduced during 7 days since admission.

The median time to surgery when demand was within the 2-day benchmark was 1.5 days (95% CI 1.4–1.5). The median time to surgery when demand required 4, 6, and 7 or more days to provide surgery was 1.6 days (95% CI 1.5–1.7), 1.7 days (95% CI 1.7–1.8), and 1.9 days (95% CI 1.8–2.0) (Table 3). After adjustment, median time to surgery was 5.1% (95% CI 4.1–6.1), 12.6% (95% CI 10.6–14.5), and 22.2% (95% CI 17.8–26.6) longer when demand required 4-, 6-, or 7 or more- days to provide surgery, compared to the 2-day benchmark.

### Patients with a medical reason for delay

**Fig. 3:**
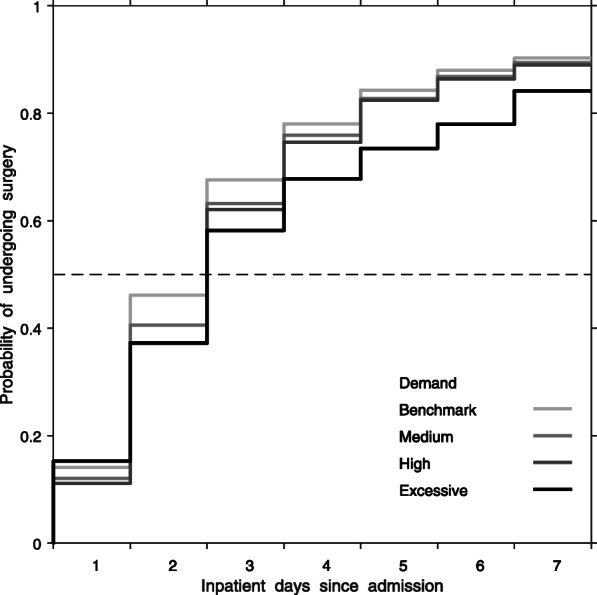
Unadjusted probability of undergoing surgery within a certain time, by demand, among those with a medical reason for delay. Benchmark demand (within 2-days), medium demand (within 4 days), high demand (within 6 days), and excessive demand (7 or more days)

Overall, 6190 of 10,144 patients (61.0%) with a medical reason for delay were admitted when demand was greater than the 2-day benchmark. For 3954 patients admitted when demand was within the 2-day benchmark, 46, 78, and 90% underwent surgery within 2, 4, and 7 days. For 4807 patients admitted during medium demand, 41, 76, 89% underwent surgery within 2, 4, and 7 days. For 1206 patients admitted during high demand, 37, 75, and 89% underwent surgery within 2, 4, and 7 days. For 177 patients admitted during excessive demand, 37, 68, and 84% underwent surgery within 2, 4, and 7 days (Fig. 3). The percentage change between excessive and benchmark demand remained similar during 7 days since admission.

The median time to surgery when demand was within the 2-day benchmark was 2.2 days (95% CI 2.1–2.4). The median time to surgery when demand required 4, 6, and 7 or more days to provide surgery was 2.4 days (95% CI 2.3–2.5), 2.5 days (95% CI 2.4–2.7), and 2.7 days (95% CI 2.2–3.1), (Table 3). After adjustment, median time to surgery was 5.6% (95% CI 2.0–9.3), 9.1% (95% CI 3.0–15.2), and 20.0% (95% CI 1.8–38.1) longer when demand required 4-, 6-, or 7 or more- days to provide surgery, compared to the 2-day benchmark.

## Discussion

### Main findings

We sought to determine the extent to which various levels of demand contributed to inappropriate surgical delays after a hip fracture. The absolute median time to surgery was 1.5 days and 1.9 days when demand required 2 days and 7 or more days respectively to provide surgery. Compared to demand that could be served within 2 days, the adjusted median time to surgery was 22% longer when demand for hip fracture surgery was highest. This percentage change was similar for those with and without medical reasons that could delay hip fracture surgery.

### Comparisons with other studies

Several strategies are proposed to reduce health declines among patients awaiting scheduled surgeries including triage based on self-reported health and expanding surgical capacity [22]. Fewer strategies are proposed for those admitted for non-scheduled surgeries. Balancing capacity to match varying demand is complex with the need for surgical services to expand where required to satisfy increases in demand [23]. Planning requires knowledge of variation in demand (both in terms of time of presentation and the number of patients presenting) to inform extra capacity for non-scheduled surgeries [24]. This planning also requires acknowledgement from hospital administrators that a mismatch between demand and capacity, resulting in either unused capacity when supply exceeds demand or a delay when demand exceeds supply, is possible [25]. However, overall patient –, process –, and cost – outcomes favor periods of unused capacity to maximise the proportions of patients surgically-treated earlier [10].

McIsaac and colleagues indicated the availability of operating rooms and surgeons led to delayed access to non-scheduled surgeries for 38.5% of patients [10]. For many sites, scheduled and non-scheduled surgeries are organised separately. If these surgeries were better integrated, capacity could be drawn away temporarily from scheduled surgeries to meet short term increased demand in non-scheduled surgeries. Given the longer recommended benchmarks for surgery of scheduled procedures, the outcomes may be less affected by delays lasting a few days (whereas such delays would be critical in the non-scheduled) [26].

However, integrating the organisation of scheduled and non-scheduled surgeries is complex. For example, extra operating room capacity allocated for non-scheduled procedures is often poorly protected at higher volume sites due to pressures to meet waiting times for scheduled procedures [27]. Others propose the addition of a dedicated operating room for non- scheduled procedures to optimise variation in demand and capacity [28]. However, this has been shown to lead to resource waste at low volume sites [28]. Alternatively, variation in demand for non-scheduled cases may be mitigated by a single-entry model whereby schedule cases are guaranteed for a given day irrespective of the potential need to prioritize non-scheduled cases before them [29].

The optimal supply of surgeons is potentially more complex – accommodating for variation in demand while ensuring sufficient volume to optimise care quality [30]. Indeed, high volume surgeons with appropriate specialization are associated with improved patient outcome when compared to low volume surgeons with general specialization [31]. This may be addressed by allocating additional capacity for non-scheduled services to higher volume sites with agreements to accept patients from neighbouring lower volume sites when their demand exceeds supply [32].

Excessive demand impacts access to surgical procedures as well as access to medical interventions required to prepare patients for these procedures. This was supported by the current study whereby the median time to surgery was almost 1 day longer for patients with a medical reason for delay when compared to patients without a medical reason for delay across all demand categories. Indeed, up to 7% of unscheduled patients may require medical intervention preoperatively [17]. It is not clear whether delaying surgery for medical interventions is beneficial or harmful for patients after hip fracture. A recent systematic review sought to determine whether the association between time to hip fracture surgery and outcomes varied across subgroups requiring medical treatments [33]. Anticoagulants were more common in patients who were delayed to surgery than patients who were not delayed to surgery in most studies included in the review [33]. However, no study formally assessed the association between time to surgery and mortality among patients receiving anticoagulant therapy and those not receiving anticoagulant therapy [33]. The review identified no additional studies which assessed the timing-outcome association among patients requiring medical treatment preoperatively [33].

### Limitations

It is well established that delays to surgery after hip fractures are associated with perioperative complications, inhospital death, and death within 12 months [6, 7, 33]. The factors responsible for these delays are less well understood [34]. There is therefore a need to identify modifiable factors which delay access to surgery. Here we reported an association between one such factor - demand and time to hip fracture surgery. We noted a small absolute difference between those admitted when demand was lowest and those admitted when demand was at its highest. We did not explore other potentially modifiable factors which may lead to delays to hip fracture surgery as these data were not available. Further, we did not report whether delays subsequent to excess demand influence the occurrence of postoperative complications and death.

We were constrained by information available from the CIHI with limited variables for adjustment (e.g. prefracture function). We were unable to quantify demand by clearance time in hours, or time to surgery in hours. This may lead to an underestimation of the association between demand and surgical timing. We employed the maximum weekly service rate as the denominator for demand; reported associations may vary for lesser weekly service rates. We examined the effect of competing demand among patients with a hip fracture. However, such patients may also compete for operative resources with other scheduled and non-scheduled patients. We did not have data to quantify this demand for all surgical procedures which may vary across sites. Therefore, we employed a random effects model to allow the effect of demand on timing to vary by hospital. We excluded 2437 patients surgically-treated in a hospital with an annual volume of 24 surgeries or less. This included all patients surgically-treated in the Territories. Further, we did not have data from Quebec. This limits the findings in those jurisdictions. Finally, for patients admitted when demand was within the 2-day benchmark only 68% underwent surgery within the recommended 2 days. This is lower than CIHI’s reported 86% for 2020 [35]. This may be due in part to the timeframe of data capture (2004–2012) as well as the measure of time in days.

## Conclusions

Excessive demand is associated with up to a 22% increase in the time needed for half of hip fracture patients to undergo surgery. This increase was observed for patients presenting with and without medical reasons that could delay hip fracture surgery after adjustment. Such delays could potentially be mitigated through better anticipation of day-to-day supply and demand coupled with increased resource capability.

## Supplementary information

**Additional file 1: Supplementary file 1**. Plan of Analysis.

## Data Availability

We studied patient records that were anonymised and de-identified by a third party, the Canadian Institute for Health Information, an organization that provides researchers access to data on Canadian residents. Data are available from the Canadian Institute for Health Information for researchers who meet the criteria for access to confidential data.

## Abbreviations

CIHI: Canadian Institute for Health Information
CI: confidence interval
NICE-124: National Institute for Health and Care Excellence guideline-124
SCU: Specialist-care-unit
