# Supplementary material for: Variation in surgical demand and time to hip fracture repair: a Canadian database study
Source: BMC Health Serv Res. 2020 Oct 10;20:935. doi: 10.1186/s12913-020-05791-5 (PMC7547438; doi:10.1186/s12913-020-05791-5)
Supplement: Supplementary file 1 — Additional file 1: Supplementary file 1. Plan of Analysis. [file 12913_2020_5791_MOESM1_ESM.docx]

# Supplementary FILE 1: Plan of Analysis

## Name of the study

Time to Hip Fracture Repair as a Function of Variation in Surgical Demand: a Canadian Database Study

## Journal

Osteoporosis International

## Plan of analysis authors

Boris Sobolev*, Jason D. Kim, Lisa Kuramoto, for The Canadian Collaborative Study on Hip Fractures

*corresponding author for plan of analysis:
Boris Sobolev
School of Population and Public Health, University of British Columbia, Vancouver, Canada
boris.sobolev@ubc.ca

## Primary study question

Does time to hip fracture surgery differ by demand, after adjusting for patient, injury, and care characteristics?

## Secondary study question

Does time to hip fracture surgery differ by demand among patients with a medical reason for delay and among those without, after adjusting for patient, injury, and care characteristics?

## Study objective

Among hip fracture patients surgically treated in Canada (excluding Quebec), between January 1, 2004 and December 31, 2012, the objectives of this study are to:

1. Estimate the distribution of time to surgery by demand described as clearance time.
2. Estimate the median time to surgery for the study population.
3. Estimate the relative difference in median time to surgery when demand requires more days than the benchmark to provide surgery.
4. Estimate the relative difference in median time to surgery when demand requires more days than the benchmark to provide surgery among patients with a medical reason for delay and among those without.

## PECO statement

Population: Patients surgically treated for non-pathological first hip fracture in Canada between 2004 and 2012

Exposure: Demand required 4-, 6-, or 7 or more- days to provide surgery

Comparator: Demand expected to be served within the 2-day benchmark

Outcome: Time to surgery

## Methodology

### Descriptive statistics

Study cohort

1. Describe patient, injury, and care characteristics, overall and by demand. Report frequency and percentage of patients for each level of the variable (Table 1).

Time to surgery

1. Estimate the median time to surgery by demand.
   - Estimate the cumulative probabilities of surgery within a certain time from admission by demand. We treat times to surgery as right-censored observations if performed after seven inpatient days, and as interval-censored observations if performed on any other inpatient day. We estimate the cumulative probabilities of surgery using a non-parametric method for interval-censored data (via SAS’ Proc ICLIFETEST procedure).
   - Report the distribution of time to surgery by demand (Figure 1).
   - Estimate the median time to surgery by demand. The median time to surgery is the time when the cumulative probability of surgery first equals or exceeds 0.5.
   - Report the median times to surgery by demand and their 95% CIs (Table 2)

### Wait time for surgery when clearance time exceeds the 2-day access policy

1. Estimate the unadjusted relative difference in median time to surgery (days) between patients admitted when demand expected to exceed the 2-day benchmark and patients admitted when demand expected to be served within the benchmark.
   - Estimate the unadjusted relative difference in the median time to surgery using interval regression (via Stata’s *meintreg* command). We treat times to surgery as right-censored observations if performed after seven inpatient days, and as interval-censored observations if performed on any other inpatient day. We will model the logarithm of time to surgery as a function of demand as indicator variables.
   - We interpret the exponential of the regression coefficient for the study variable as the unadjusted relative difference in median time to surgery when demand requires more days than the benchmark to provide surgery compared to when demand expected to be served within the 2-day benchmark. For a positive regression coefficient, (exp(regression coefficient)-1)*100% is the percentage increase in time to surgery when demand requires more days than the benchmark.
   - Report the unadjusted relative difference in time to surgery and 95% CI (Table 2).
2. Estimate the adjusted relative difference in median time to surgery (days) between patients admitted when demand expected to exceed the 2-day benchmark and patients admitted when demand expected to be served within the benchmark.
   - Estimate the adjusted relative difference in the median time to surgery using two-level interval regression (via Stata’s *meintreg* command). We treat times to surgery as right-censored observations if performed after seven inpatient days, and as interval-censored observations if performed on any other inpatient day. We will model the logarithm of time to surgery as a function of demand as indicator variables by level of surgical hospital. We group patients by hospital ID and allow the effect of demand to vary by hospital. We specify random intercepts for hospital ID and random coefficients for the demand variable. We account for intra-hospital correlation using *vce(cluster hospsx)*.
   - Adjust for age, sex, prefracture health status, timing of admission, admission status, preoperative transfer history, preoperative procedures, medical reason for delay, hospital type at surgery, fracture type, procedure type, treatment era, and province. Exclude patients with unknown sex.
   - We interpret the exponential of the regression coefficient for the study variable as the adjusted relative difference in median time to surgery when demand requires more days than the benchmark to provide surgery compared to when demand expected to be served within the 2-day benchmark. For a positive regression coefficient, (exp(regression coefficient)-1)*100% is the percentage increase in time to surgery when demand requires more days than the benchmark.
   - Report the adjusted relative difference in time to surgery and 95% CI (Table 2).

## Subgroup analysis

1. Repeat steps 3 to 4 among those with and without a medical reason for delay (Figures 2–3; Tables 3–4).

## Tables

1. **Table 1.** Characteristics of XXX,XXX patients who underwent first hip fracture surgery in Canada, 2004–2012, overall and by demand.
2. **Table 2.** Time to hip fracture surgery by demand.
3. **Table 3.** Time to hip fracture surgery by demand, among those without a medical reason for delay. [similar to Table 2 template]
4. **Table 4.** Time to hip fracture surgery by demand, among those with a medical reason for delay. [similar to Table 2 template]

## Figures

1. **Figure 1**. Crude probability of undergoing surgery within a certain time, by demand. Demand is measured by clearance time, the expected length of time for all hospitalized hip fracture patients on day of index patient admission to undergo surgery at maximum weekly service rate of the surgical hospital in the corresponding fiscal quarter.
   - x-label: Time since admission (days)
   - y-label: Probability of undergoing surgery
   - Legend: Demand: 2 days, 4 days, 6 days, 7 or more days.
   - Include dashed horizontal line at probability value of 0.5. Included dashed vertical lines from 0.5 down to the median time.
2. **Figure 2**. Crude probability of undergoing surgery within a certain time, by demand, among those without a medical reason for delay. Demand is measured by clearance time, the expected length of time for all hospitalized hip fracture patients on day of index patient admission to undergo surgery at maximum weekly service rate of the surgical hospital in the corresponding fiscal quarter.
   - x-label: Time since admission (days)
   - y-label: Probability of undergoing surgery
   - Legend: Demand: 2 days, 4 days, 6 days, 7 or more days.
   - Include dashed horizontal line at probability value of 0.5. Included dashed vertical lines from 0.5 down to the median time.
3. **Figure 3**. Crude probability of undergoing surgery within a certain time, by demand, among those with a medical reason for delay. Demand is measured by clearance time, the expected length of time for all hospitalized hip fracture patients on day of index patient admission to undergo surgery at maximum weekly service rate of the surgical hospital in the corresponding fiscal quarter.
   - x-label: Time since admission (days)
   - y-label: Probability of undergoing surgery
   - Legend: Demand: 2 days, 4 days, 6 days, 7 or more days.
   - Include dashed horizontal line at probability value of 0.5. Included dashed vertical lines from 0.5 down to the median time.

## Table templates

**Table 1.** Characteristics of XXX,XXX patients who underwent first hip fracture surgery in Canada, 2004–2012, overall and by demand.

|  |  | | Demand*; no. (%) of patients | | | | | | | |
| --- | --- | --- | --- | --- | --- | --- | --- | --- | --- | --- |
|  | All patients (N=151,952) | | 2 days (N=61,090) | | 4 days (N=71,183) | | 6 days (N=17,192) | | 7 or more days  (N=2,487) | |
|  | n | (%) | n | (%) | n | (%) | n | (%) | n | (%) |
| Age, years |  |  |  |  |  |  |  |  |  |  |
| 65–74 |  |  |  |  |  |  |  |  |  |  |
| 75–84 |  |  |  |  |  |  |  |  |  |  |
| 85–94 |  |  |  |  |  |  |  |  |  |  |
| ≥95 |  |  |  |  |  |  |  |  |  |  |
| Sex† |  |  |  |  |  |  |  |  |  |  |
| Women |  |  |  |  |  |  |  |  |  |  |
| Men |  |  |  |  |  |  |  |  |  |  |
| Prefracture health: from‡ |  |  |  |  |  |  |  |  |  |  |
| Home without comorbidity |  |  |  |  |  |  |  |  |  |  |
| Home with comorbidity or home care |  |  |  |  |  |  |  |  |  |  |
| Facility |  |  |  |  |  |  |  |  |  |  |
| Elsewhere |  |  |  |  |  |  |  |  |  |  |
| Timing of admission |  |  |  |  |  |  |  |  |  |  |
| Weekday 12 am to 3:59 pm |  |  |  |  |  |  |  |  |  |  |
| Weekday 4 pm to 11:59 pm |  |  |  |  |  |  |  |  |  |  |
| Weekend |  |  |  |  |  |  |  |  |  |  |
| Admission status |  |  |  |  |  |  |  |  |  |  |
| Urgent/Emergent |  |  |  |  |  |  |  |  |  |  |
| Otherwise |  |  |  |  |  |  |  |  |  |  |
| Preoperative transfer history |  |  |  |  |  |  |  |  |  |  |
| No |  |  |  |  |  |  |  |  |  |  |
| Yes |  |  |  |  |  |  |  |  |  |  |
| Preoperative procedures |  |  |  |  |  |  |  |  |  |  |
| No |  |  |  |  |  |  |  |  |  |  |
| Yes |  |  |  |  |  |  |  |  |  |  |
| Medical reason for delay§ |  |  |  |  |  |  |  |  |  |  |
| No |  |  |  |  |  |  |  |  |  |  |
| Yes |  |  |  |  |  |  |  |  |  |  |
| Hospital type at surgery\|\| |  |  |  |  |  |  |  |  |  |  |
| Teaching |  |  |  |  |  |  |  |  |  |  |
| Community-Large |  |  |  |  |  |  |  |  |  |  |
| Community-Medium, Small |  |  |  |  |  |  |  |  |  |  |
| Fracture type |  |  |  |  |  |  |  |  |  |  |
| Transcervical |  |  |  |  |  |  |  |  |  |  |
| Intertrochanteric or subtrochanteric |  |  |  |  |  |  |  |  |  |  |
| Procedure type |  |  |  |  |  |  |  |  |  |  |
| Fixation |  |  |  |  |  |  |  |  |  |  |
| Arthroplasty |  |  |  |  |  |  |  |  |  |  |
| Treatment era |  |  |  |  |  |  |  |  |  |  |
| 2004–2006 |  |  |  |  |  |  |  |  |  |  |
| 2007–2009 |  |  |  |  |  |  |  |  |  |  |
| 2010–2012 |  |  |  |  |  |  |  |  |  |  |
| Province of surgery |  |  |  |  |  |  |  |  |  |  |
| Alberta |  |  |  |  |  |  |  |  |  |  |
| British Columbia |  |  |  |  |  |  |  |  |  |  |
| Manitoba |  |  |  |  |  |  |  |  |  |  |
| New Brunswick |  |  |  |  |  |  |  |  |  |  |
| Newfoundland and Labrador |  |  |  |  |  |  |  |  |  |  |
| Nova Scotia |  |  |  |  |  |  |  |  |  |  |
| Ontario |  |  |  |  |  |  |  |  |  |  |
| Prince Edward Island |  |  |  |  |  |  |  |  |  |  |
| Saskatchewan |  |  |  |  |  |  |  |  |  |  |

*Measured by clearance time, the expected length of time for all hospitalized hip fracture patients on day of index patient admission to undergo surgery at maximum weekly service rate of the surgical hospital in the corresponding fiscal quarter.

†For XX patients, sex was unknown.
‡Comorbidities included heart failure, chronic obstructive pulmonary disorder, ischemic heart disease (acute and chronic), dysrhythmias, hypertension, diabetes, and cancer (breast–female, prostate, renal, lung, multiple myeloma, and metastatic cancer) identified by diagnostic codes from all hospitalizations in 1 year prior to index admission, and cancer and Paget’s disease, identified by diagnostic codes from all hospitalizations during the hip fracture care episode.

§At least one of the following NICE-124 conditions: anaemia, anticoagulation reversal, volume depletion, electrolyte imbalance, uncontrolled diabetes, uncontrolled heart failure, correctable cardiac arrhythmia, correctable cardiac ischaemia, acute chest infection, and exacerbation of chronic chest condition; or preoperative admission to SCU.

||For XXXX patients, hospital type was unavailable.

**Table 2**. Time to hip fracture surgery by demand.

| Demand* | No. of Surgeries | Median Time to Surgery, Days  (95% CI) | Unadjusted Percentage  Change  (95% CI) | Adjusted Percentage  Change  (95% CI)†‡ |
| --- | --- | --- | --- | --- |
| 2 days |  |  | Reference | Reference |
| 4 days |  |  |  |  |
| 6 days |  |  |  |  |
| 7 or more days |  |  |  |  |

*Measured by clearance time, the expected length of time for all hospitalized hip fracture patients on day of index patient admission to undergo surgery at maximum weekly service rate of the surgical hospital in the corresponding fiscal quarter.

†Adjusted for age, sex, prefracture health status, timing of admission, admission status, preoperative transfer history, preoperative procedures, medical reason for delay, hospital type at surgery, fracture type, procedure type, treatment era, and province.

‡Random intercepts by hospital ID and random coefficients for demand; random effects by grouping patients according to hospital ID and allowing the effect of demand to vary by the grouping structure.
